# Heterogeneity of Starved Yeast Cells in IF_1_ Levels Suggests the Role of This Protein *in vivo*

**DOI:** 10.3389/fmicb.2022.816622

**Published:** 2022-03-23

**Authors:** Kseniia V. Galkina, Valeria M. Zubareva, Nataliia D. Kashko, Anna S. Lapashina, Olga V. Markova, Boris A. Feniouk, Dmitry A. Knorre

**Affiliations:** ^1^A. N. Belozersky Institute of Physico-Chemical Biology, Lomonosov Moscow State University, Moscow, Russia; ^2^Faculty of Bioengineering and Bioinformatics, Lomonosov Moscow State University, Moscow, Russia; ^3^Department of Biological Chemistry, Sechenov First Moscow State Medical University, Moscow, Russia

**Keywords:** ATPase, stationary phase, heterogeneity, IF1, mitochondria, F_O_F_1_, starvation recovery

## Abstract

In mitochondria, a small protein IF_1_ suppresses the hydrolytic activity of ATP synthase and presumably prevents excessive ATP hydrolysis under conditions of energy deprivation. In yeast *Saccharomyces cerevisiae*, IF_1_ homologs are encoded by two paralogous genes: *INH1* and *STF1*. *INH1* expression is known to aggravate the deleterious effects of mitochondrial DNA (mtDNA) depletion. Surprisingly, no beneficial effects of *INH1* and *STF1* were documented for yeast so far, and the functions of *INH1* and *STF1* in wild type cells are unclear. Here, we put forward a hypothesis that *INH1* and *STF1* bring advantage during the fast start of proliferation after reentry into exponential growth from post-diauxic or stationary phases. We found that yeast cells increase the concentration of both proteins in the post-diauxic phase. Post-diauxic phase yeast cells formed two subpopulations distinct in Inh1p and Stf1p concentrations. Upon exit from the post-diauxic phase cells with high level of Inh1-GFP started growing earlier than cells devoid of Inh1-GFP. However, double deletion of *INH1* and *STF1* did not increase the lag period necessary for stationary phase yeast cells to start growing after reinoculation into the fresh medium. These results point to a redundancy of the mechanisms preventing uncontrolled ATP hydrolysis during energy deprivation.

## Introduction

Oxidative phosphorylation (OxPhos) is one of the major mechanisms of energy conversion in living cells (Wilson, 2017). During OxPhos, respiratory chain enzymes generate the transmembrane difference of electrochemical proton potential (Δ*μ˜*_H_^+^) that powers H^+^-transport through F_O_F_1_ ATP-synthase coupled to synthesis of ATP from ADP and inorganic phosphate. When Δ*μ˜*_H_^+^ decreases below the thermodynamic threshold for ATP synthesis, F_O_F_1_ activity reverses and the enzyme works as an ATP-driven proton pump and generates Δ*μ˜*_H_^+^ (Zubareva et al., 2020). Surprisingly, complete dissipation of Δ*μ˜*_H_^+^ often leads to inhibition of F_O_F_1_ ATPase activity. In mitochondria, this inhibition is mediated by a small protein IF_1_ that binds to F_1_ subcomplex of F_O_F_1_ (Pullman and Monroy, 1963; Satre et al., 1975) and blocks ATP hydrolysis in de-energized mitochondria. IF_1_ is believed to prevent the depletion of ATP and cell death under energy deprivation conditions (Campanella et al., 2008). In animal mitochondria, IF_1_ plays an important role in the assembly of F_O_F_1_ (He et al., 2018), inhibits autophagy (Campanella et al., 2009), and is involved in the formation of mitochondrial cristae (Weissert et al., 2021). Since cancer cells usually proliferate in poorly aerated environments, they upregulate IF_1_ expression: an increase in IF_1_ is an important prognostic factor of tumor development (Galber et al., 2020).

Yeast *Saccharomyces cerevisiae* harbor two genes encoding IF_1_ homologs: *INH1* and *STF1* (Ichikawa et al., 1990). In the presence of protonophores, mitochondria isolated from the yeast strain with double deletion of IF_1_ genes, *Δinh1Δstf1*, hydrolyze ATP much faster than the mitochondria of wild type yeast do (Ichikawa et al., 1990; Venard et al., 2003). Moreover, deletion of IF_1_ homolog increased ATP hydrolysis rate in another yeast species, *Ustilago maydis* (Lucero et al., 2021).

At the same time, Inh1p protein plays a detrimental role in the metabolism of *rho^0^ S. cerevisiae* cells that lack mitochondrial DNA (mtDNA) and are incapable of OxPhos. Since three subunits of the ATP-synthase F_O_-subcomplex are encoded in mtDNA, *rho^0^* cells assemble only the F_1_-subcomplex in the matrix. In *rho^0^* cells, futile ATP hydrolysis by F_1_ is coupled with the electrogenic exchange of matrix ADP^3−^ to cytosolic ATP^4−^ mediated by adenine nucleotide carriers. Inhibition of F_1_-subcomplex ATPase activity by Inh1p decreases Δ*μ˜*_H_^+^ in *rho^0^* yeast mitochondria, whereas mitochondrial Δ*μ˜*_H_^+^ is essential for *rho^0^* cells proliferation. Indeed, increased *INH1* expression decreases the growth rate of *rho^0^* yeast cells, while *INH1* deletion accelerates growth (Liu et al., 2021). Furthermore, while yeast cells with deleted i-AAA protease gene *YME1* cannot survive without mtDNA, the *Δyme1Δinh1 rho^0^* strain proved to be viable. It is suggested that deletion of *YME1* suppresses Inh1p degradation, increases Inh1p concentration in the mitochondrial matrix and, therefore, prevents ATP hydrolysis in *rho^0^* cells (Kominsky et al., 2002). However, the *INH1* deletion phenotypes discussed above do not clarify its physiological role in wild type yeast cells. On the contrary, the examples show that the presence of a functional *INH1* gene is detrimental under conditions when the respiratory chain activity is low.

*INH1* or *STF1* genes were also identified in several high-throughput screenings. For instance, *Δinh1* strain was found to be highly susceptible to propionic acid stress (Mira et al., 2009) and incapable of filamentous growth (Jin et al., 2008). *STF1* deletion increased the survival of yeast cells during prolonged starvation in synthetic medium (Garay et al., 2014). These results imply that the deletion of ATPase inhibitor proteins can manifest at the level of the whole cells, but these results were not verified with independently obtained mutants.

Taken together, these works show that information about the biological role of IF_1_-like proteins is largely limited to (1) experiments on isolated mitochondria; (2) the results of genetic screenings; or (3) deleterious effects of these proteins that reduce the growth rate of intact *rho^0^* cells. In our work, we attempted to get an insight into the physiological (adaptive) role of Inh1p and Stf1p in yeast cells. We assessed the expression of these proteins under different yeast growth conditions. We also tested how Inh1p level in post-diauxic phase yeast cells is correlated with the chance to proliferate within the first few hours after inoculation into a rich growth medium. Finally, we investigated the phenotype of *INH1* and *STF1* genes double deletion in a variety of stressful conditions, e.g., in the stationary phase and in the presence of protonophores.

## Materials and Methods

### Yeast Strains, Growth Conditions, and Reagents

Yeast strains used in the study are listed in Supplementary Table S1. Deletion, prototrophy, and GFP-fusion strains were obtained by homologous recombination of the PCR product with heterologous selection markers. All newly generated strains were verified by PCR with primers annealed to DNA regions outside the disruption cassette (Supplementary Table S2; Supplementary Figure S1). Deletions of *INH1* and *STF1* were validated using Reverse transcription-qPCR (RT-qPCR, see below). We also verified that Stf1-GFP and Inh1-GFP expressing cells show mitochondrial localization of GFP (Supplementary Figure S2).

We prepared standard rich (yeast peptone, YP) growth media as described by Sherman (2002). We obtained peptone and yeast extract from BioSpringer, D-glucose and galactose from Helicon, raffinose from Chimmed, glycerol from Panreac, lactate from Alfa Aesar, and agar from DiaM. Before the experiments, yeast cells were grown at 30°C in 5 ml of the liquid medium in 50 ml flasks overnight up to the exponential growth phase. We also assessed yeast cells incubated in batch cultures for 2 days (post-diauxic cells) and 7–10 days (stationary phase cells).

### Flow-Cytometry and Fluorescent Microscopy

GFP fluorescence was assessed with a CytoFlex (Beckman-Coulter) flow cytometer using excitation wavelength of 488 nm and the emission filter (525/40 nm). At least 10,000 events were analyzed in each experiment. To quantify the proportion of yeast cells expressing Inh1-GFP and Stf1-GFP, we introduced the gating threshold so that 98% of control yeast cells without GFP would be below this threshold. Thereafter, we applied this threshold to all flow-cytometry data of Inh1-GFP and Stf1-GFP expressing cells. We considered events above this threshold as GFP-positive cells and below this threshold as GFP-negative cells. Acquisition and analysis were performed using CytExpert v 2.0 software; we rendered representative images using flowCore and ggcyto libraries of R programming language (Hahne et al., 2009; Van et al., 2018). The fluorescence intensity values were log-transformed using logicleTransform function of flowCore (Parks et al., 2006).

We photographed the cells expressing GFP using the fluorescence microscope Olympus BX41 with the U-MNIBA3 (excitation wavelength 470–495 nm; beamsplitter filter 505 nm; and emission 510–550 nm) filter set. Photographs were taken with a DP30BW charged-coupled device camera.

### Cell-to-Cell Heterogeneity Analysis

The strain expressing Inh1p-GFP was grown for 2 days in liquid yeast peptone dextrose (YPD) medium up to the post-diauxic or for 7 days to the stationary phase. Then, we inoculated the cells into the fresh YPD medium to the final OD_550_ = 0.1 (2 × 10^6^ cell/ml). We analyzed photographs of cells taken after 0, 2, and 4 h of incubation. We counted the number of cells with/without buds and with/without GFP. About 130–630 cells were analyzed in each sample.

### Quantitative Reverse Transcription PCR Analysis

RNA was isolated from yeast cells using the hot formamide extraction method described in Shedlovskiy et al. (2017). cDNA was synthesized by annealing 2 μg of RNA with 0.1 μg of random hexamers and 0.1 μg of Oligo-dT using Superscript III reverse transcriptase (Thermo Fisher Scientific) for 1 h at 42°C. RT-qPCR was carried out using the CFX96 Touch™ Real-Time PCR Detection System (Bio-Rad, Hercules, CA, United States). Primer sequences for quantification of *INH1* and *STF1* gene expression are listed in Supplementary Table S2. Eva Green master mix (Syntol, Russia) was used for the detection of DNA accumulation during the reaction. The thermal profile for the EVA Green RT-qPCR included an initial heat-denaturing step at 95°C for 3 min, 40 cycles at 95°C for 15 s, an annealing step for 30 s, and 72°C for 30 s, coupled with fluorescence measurements. Following amplification, the melting curves of the PCR products were monitored to determine the specificity of the amplification. Target mRNA levels were normalized to the reference gene *ACT1*.

### Isolation of Mitochondria and Respirometry

We isolated mitochondria from the WT (*HIS^+^ TRP^+^*) and *Δinh1Δstf1* strains grown in yeast peptone glycerol (YPGly) medium using the protocol described earlier by Bazhenova et al. (1998). To test whether mitochondrial preparations of wild type and *Δinh1Δstf1* strains are similar in mitochondrial protein content, we assessed the uncoupled respiration of isolated mitochondria with Clark-type oxygen electrode (Strathkelvin Instruments 782, United Kingdom) at 25°C. Measurements were performed in the mitochondria incubation medium containing 0.6 M mannitol, 10 mM Tris–HCl, 0.5 mM MgCl_2_, and 2 mM potassium phosphate (pH 7.4). We measured the rate of succinate (5 mM) oxidation and NADH-dependent respiration (8 mM pyruvate with 2 mM malate) as the substrates, to uncouple mitochondria we added 200 nM FCCP (carbonyl cyanide-p-trifluoromethoxyphenylhydrazone).

### ATP Hydrolysis Measurements

ATP hydrolysis by isolated mitochondria was registered *via* NADH oxidation in the ATP regenerating system as in Vasilyeva et al. (1980). The buffer contained 20 mM HEPES, pH 8.0, 0.65 M sorbitol, 17 mM KCl, 3 mM K_2_HPO_4_, 1 mM MgCl_2_, 1 mg/ml bovine serum albumin (BSA), 2 μM myxothiazol, 200 μM NADH, 2.5 mM phosphoenolpyruvate, 20 U/ml pyruvate kinase, and 20 U/ml lactate dehydrogenase. ATP was added to 1 mM to start the reaction. If indicated, valinomycin and nigericin were added to 500 nM each to dissipate Δ*μ˜*_H_^+^.

### Yeast Growth Analysis

Cells were diluted to the optical density of OD_550_ = 0.05 (10^6^ cell/ml) and inoculated in 100 μl of the liquid medium into a 96-well plate (Eppendorf). Plates were incubated in a spectrophotometer (SpectrostarNANO) with the following settings: orbital shaking at 500 rpm for 2 min at 30°C before measurements; measurements were performed at 5-min intervals. We compared the maximal growth rates (μ_max_) and lag-period between the control and mutant strains. To calculate growth rate μ_max_, we took log_2_-transformed OD values and fitted them with the standard linear model using R. We calculated slopes for each curve in 250 min sliding windows and took maximal values. μ_max_ here is equal to 1/duplication time.

### Determination of ATP Level in Yeast Cells

Cells grown in YPD to the post-diauxic phase (2 days) or the stationary phase (7 or 10 days) were harvested by centrifugation and resuspended in PBS buffer to the OD_550_ of about 0.1–0.2. To measure the ATP level in yeast during exponential growth, the resuspension step was omitted and ATP was extracted directly from the growing culture. The suspensions were mixed with dimethyl sulfoxide (v/v 1:9) to extract ATP (Romanova et al., 1997). ATP amount in the extracts was determined using ATP Bioluminescence Assay Kit CLS II (Roche). The resulting values were normalized to the number of yeast cells in the corresponding samples measured by flow cytometry (Beckman-Coulter).

### Competitive Assay

We mixed exponentially growing (1) WT *LEU^+^* with WT *HIS^+^TRP*^+^ *leu*^−^ and (2) WT *LEU^+^* with *Δinh1Δstf1 leu*^−^ *HIS^+^TRP*^+^ in liquid YPD medium in equal proportion (1:1) to the final concentration of 4 × 10^4^ cell/ml. Then, every day we took a part of the suspension and inoculated it into the fresh YPD medium (1:500). Each time, we plated this suspension on the SD-Leu and SD-His agar plates and after 2 days calculated the proportion of *LEU*^+^ cells in the suspension as the number of the colonies on SD-Leu divided by the total number of colonies on SD-Leu and SD-His.

## Results

### Heterogeneity of Inh1p and Stf1p Levels in Post-diauxic Yeast Cultures

**Figure 1:**
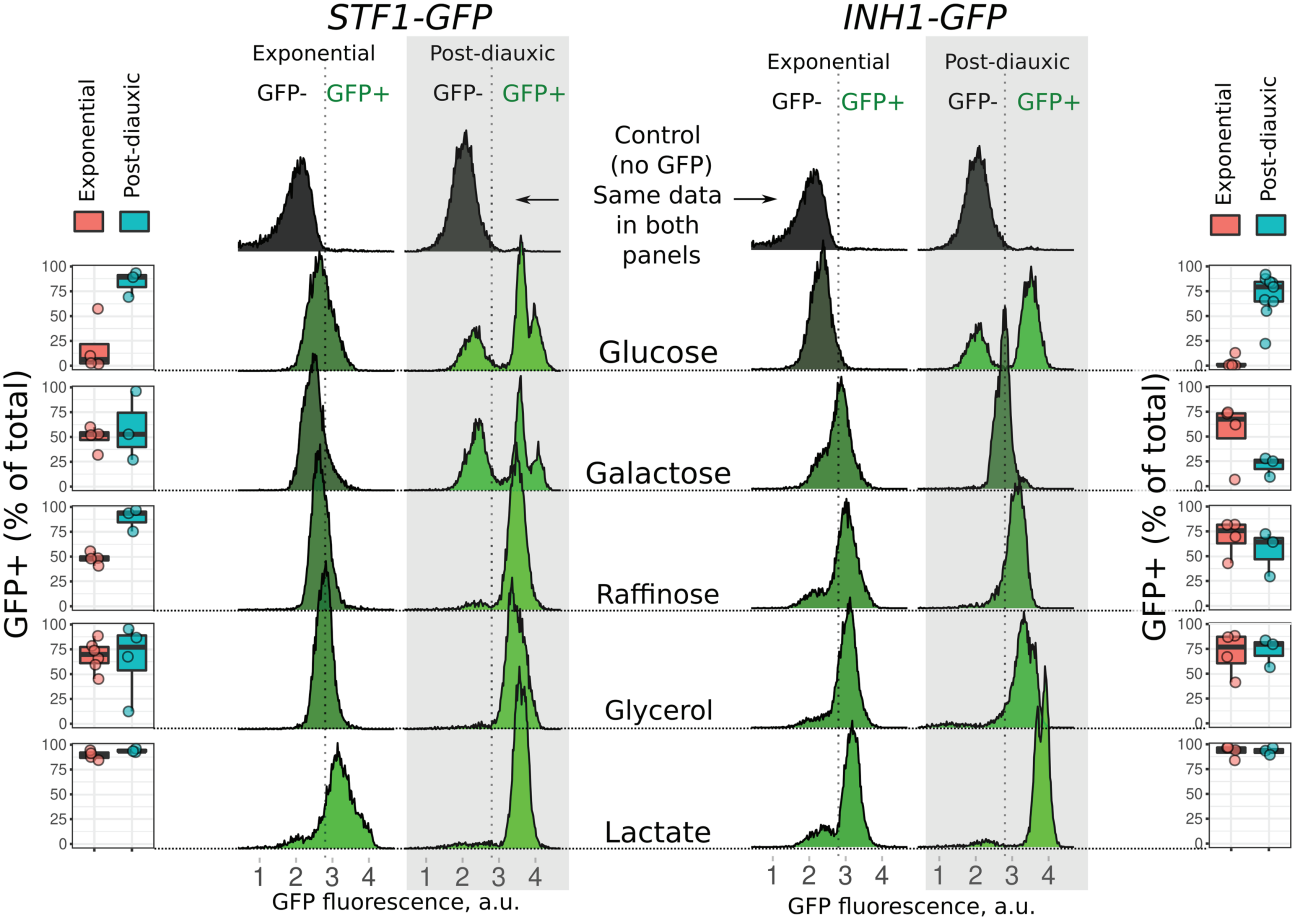
Accumulation of Stf1-GFP (left panel) and Inh1-GFP (right panel) in exponentially growing and post-diauxic *BY4741* yeast cells. Boxplots show the proportion of yeast cells with GFP levels above the autofluorescence signal. Histograms show the results of representative experiments. Upper histograms correspond to autofluorescence of the control cells without GFP which are the same for both strains.

Deletion of a gene usually produces a pronounced phenotype under the conditions that induce high expression of this gene. Therefore, before testing the effects of *INH1* and *STF1* deletion, we assessed the expression of these genes in yeast utilizing different carbon sources. We took yeast strains with chromosomal copies of the *INH1* and *STF1* genes tagged with the GFP gene (Supplementary Table S1) and analyzed them using fluorescent microscopy and flow cytometry. We assumed that C-terminal GFP tagging would not affect the inhibitory function of Inh1p or Stf1p, since they bind to F_1_ by the N-terminal domain (Robinson et al., 2013). During exponential growth in a rich medium with glucose, yeast cells maintained low levels of Stf1-GFP and Inh1-GFP. Meanwhile, the concentration of both Inh1-GFP and Stf1-GFP increased in yeast utilizing poorly fermentable carbon sources (e.g., raffinose) or non-fermentable glycerol or lactate (Figure 1). Expression of Inh1-GFP and Stf1-GFP in another strain background (*W303*) showed a similar pattern (Supplementary Figure S3).

Next, we showed that starved yeast cells increase the levels of both IF_1_-like proteins. Post-diauxic yeast *BY4741* culture showed heterogeneity in the content of Inh1-GFP and Stf1-GFP (Figure 1). We detected two subpopulations of cells that were distinct in Inh1-GFP levels in the stationary culture cultivated in a medium supplied with glucose; for Stf1-GFP post-diauxic yeast heterogeneity was also detected in galactose based growth medium. The difference in Inh1-GFP levels was not a result of genetic variation: the cultures that were grown from single colonies also showed heterogeneity in Inh1-GFP levels (Supplementary Figure S4). Inh1-GFP concentration remained high in 10-day stationary phase cells (Supplementary Figure S5). Furthermore, when we analyzed Stf1-GFP expression, stationary phase yeast cells demonstrated three peaks corresponding to cells with different Stf1-GFP accumulation levels (Figure 1). It should be noted that there was no heterogeneity in the expression levels of Stf1-GFP and Inh1-GFP if the cultures were grown in YP raffinose medium and in *W303*-based yeast strains (Supplementary Figure S3).

**Figure 2:**
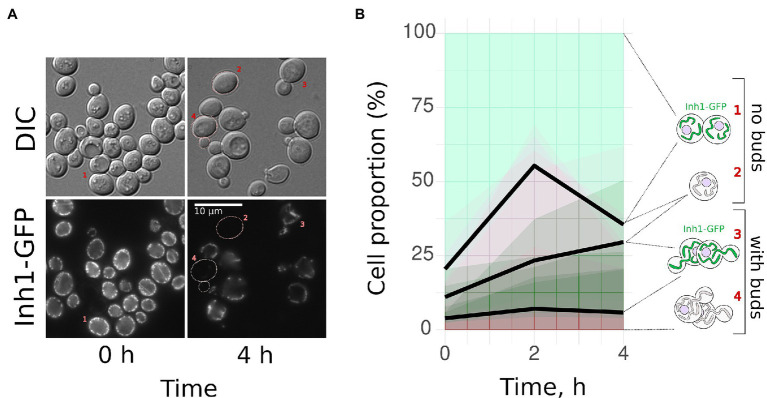
Yeast cells with Inh1-GFP start budding faster than the cells without Inh1-GFP. Post-diauxic phase yeast cells expressing Inh1-GFP were transferred into a fresh yeast peptone dextrose (YPD) medium. We observed two types of cells: those expressing Inh1-GFP and those with no detectable GFP signal. We calculated the percentages of budded vs. unbudded cells separately for these groups. **(A)** Representative photograph of the cells at the beginning of the experiment and after 4 h of growth in the fresh medium. DIC—Differential Interference Contrast; **(B)** Change in the proportion of (1) Inh1-GFP positive non-budding; (2) Inh1-GFP negative non-budding; (3) Inh1-GFP positive budding; and (4) Inh1-GFP negative budding cells. The average proportions are shown as black lines, the results of the individual experiments are illustrated with semi-transparent green or red areas (data of five separate experiments, 369 cells on average in each data point). The number of budding cells with GFP (category 3) increased significantly more than the number of budding cells without GFP (category 4); *p* = 0.016 according to Wilcoxon rank sum test (*n* = 5).

Given that *BY4741*-based yeast cells in the post-diauxic phase had two subpopulations (with and without Inh1-GFP), we decided to trace separately the cells from these subpopulations after transfer to a fresh medium. We suggested that the cells with high Inh1-GFP levels begin to form buds faster than the cells without Inh1-GFP. To test this hypothesis, we inoculated stationary phase *BY4741* Inh1-GFP cells into the fresh YPD medium and photographed them immediately or after 2 or 4 h after the transfer. We counted the cells belonging to one of the four categories: (1) unbudded cells with Inh1-GFP; (2) unbudded cells without detectable GFP signal; (3) budding cells with Inh1-GFP; and (4) budding cells without detectable GFP signal. Figure 2A shows representative photographs of all these categories. On average, 16.6 ± 6.8% (mean + SE) Inh1-GFP-positive cells started budding during the first 4 h of growth in a fresh medium, while only 2.0 ± 1.4% (mean + SE) Inh1-GFP-negative cells started budding during this period (Figure 2B).

### Genetic and Biochemical Verification of *INH1* and *STF1* Double Deletion

**Figure 3:**
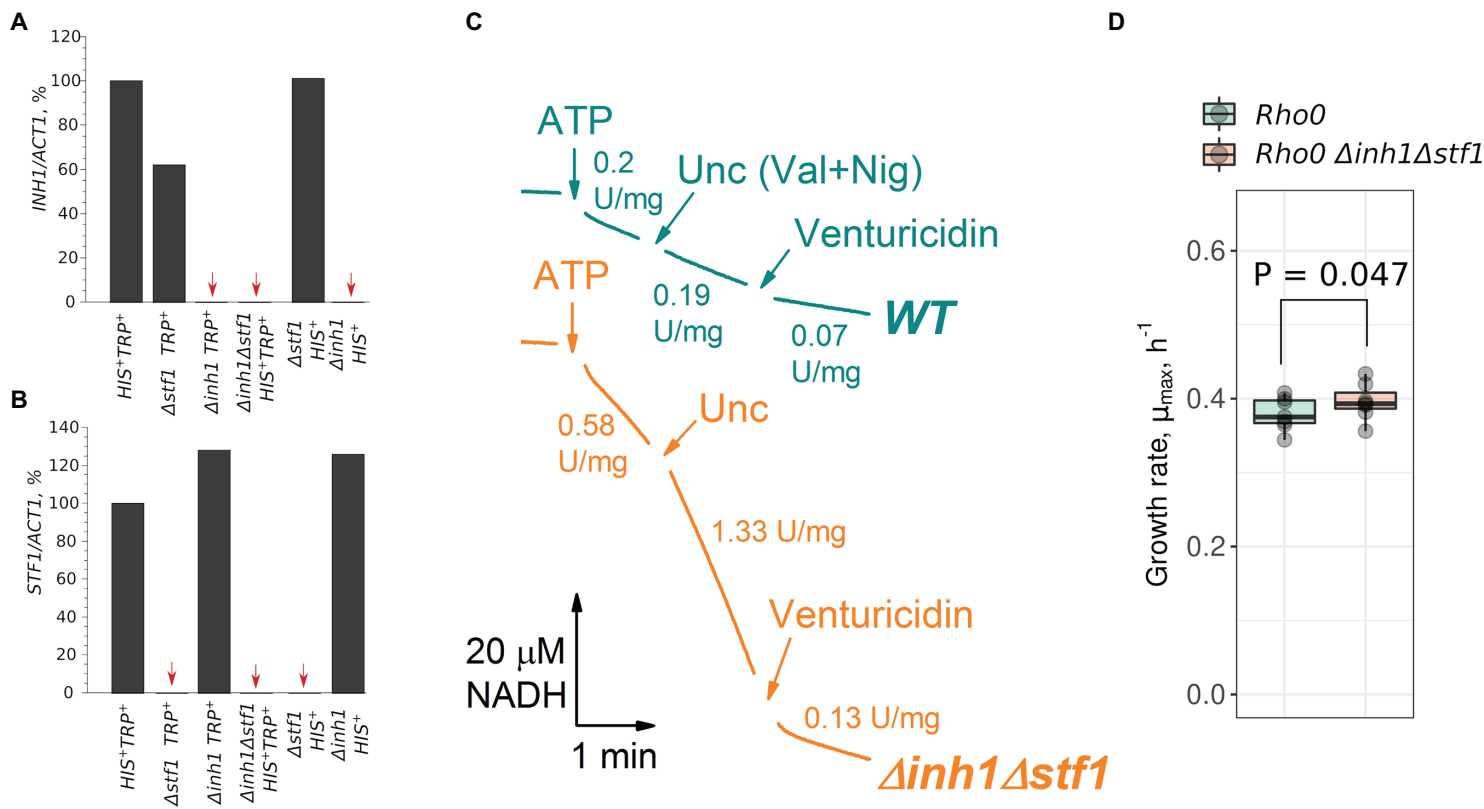
Double deletion of *INH1* and *STF1* increases the rate of ATP hydrolysis by isolated mitochondria and the growth rate of cells without mitochondrial DNA (mtDNA; *rho^0^*). **(A,B)** *INH1* and *STF1* mRNA levels in the control and deletion strains. All mRNA levels were normalized to the *ACT1* mRNA level, and the value in control (*HIS^+^ TRP*^+^) cells was set at 100%. Red arrows indicate strains with undetectable mRNA levels (single experiment); **(C)** ATP hydrolysis rate by mitochondria isolated from the control (WT) and *Δinh1Δstf1* cells. ATP hydrolysis was registered as NADH oxidation in ATP regenerating system (see Materials and Methods) in the presence of respiratory chain inhibitor myxothiazole. The reaction was started by addition of ATP to 1 mM. When indicated (Unc, Val + Nig), a mixture of valinomycin and nigericin was added to dissipate Δμ_H_^+^.; Venturicidin was added to reveal F_O_F_1_-specific activity; representative curves of two independent mitochondrial isolations; **(D)** Growth rates (μ_max_) of *rho^0^* cells lacking mtDNA derived from the control (WT) and *Δinh1Δstf1* strains in rich medium supplemented with glucose (YPD). *p* value was calculated according to paired Wilcoxon signed-rank test.

Although Inh1p and Stf1p proteins have pronounced differences in amino acid sequence and in pH dependency of their inhibitory activity (Cabezon et al., 2002), their functions may overlap. Therefore, to test the phenotypes associated with IF_1_-like proteins deficiency, we produced a strain in which both *INH1* and *STF1* were deleted. We also produced a control strain with the same set of prototrophic marker genes (Supplementary Table S1). Using qRT-PCR, we found no detectable expression of *INH1* and *STF1* in the double deletion strain (Figures 3A,B). Moreover, mitochondria isolated from the *Δinh1Δstf1* strain demonstrated an increased rate of ATP hydrolysis which, in contrast to the wild type mitochondria, was further stimulated by uncoupling (Figure 3C). Importantly, ATPase activity normalized to succinate oxidation rate in uncoupled wild type mitochondria was lower (0.27 ± 0.10, ATP hydrolyzed/O_2_ consumed; mean ± SD) than in uncoupled mutant mitochondria (2.69 ± 1.94, ATP hydrolyzed/O_2_ consumed; mean ± SD). Similar results we obtained when we normalized the ATPase activity to respiration rate in the presence of NADH-dependent substrate (pyruvate + malate): the ratio of the activities was 0.57 ± 0.31 (ATP hydrolyzed/O_2_ consumed; mean ± SD) for the wild type mitochondria, while in *Δinh1Δstf1* mitochondria it was 3.68 ± 1.45 (ATP hydrolyzed/O_2_ consumed; mean ± SD). Using Blue Native PAGE, we assessed the relative level of F_O_F_1_ in two mitochondrial preparations of each strain. The amount of F_O_F_1_ in the *Δinh1Δstf1* mutant did not exceed that in the wild type mitochondria (Supplementary Figure S6). Therefore, the high ATP hydrolysis rate of *Δinh1Δstf1* mitochondria cannot be explained by a mere increase in F_O_F_1_ concentration. Together, these results are in good agreement with earlier experiments on *Δinh1Δstf1* yeast mitochondria (Venard et al., 2003). Finally, in line with previous studies (Liu et al., 2021), *Δinh1Δstf1* strain with depleted mtDNA (*Δinh1Δstf1 rho^0^*) demonstrated an increase in the proliferation rate compared to the control *rho^0^* strain, although the amplitude of the effect was small (Figure 3D).

**Figure 4:**
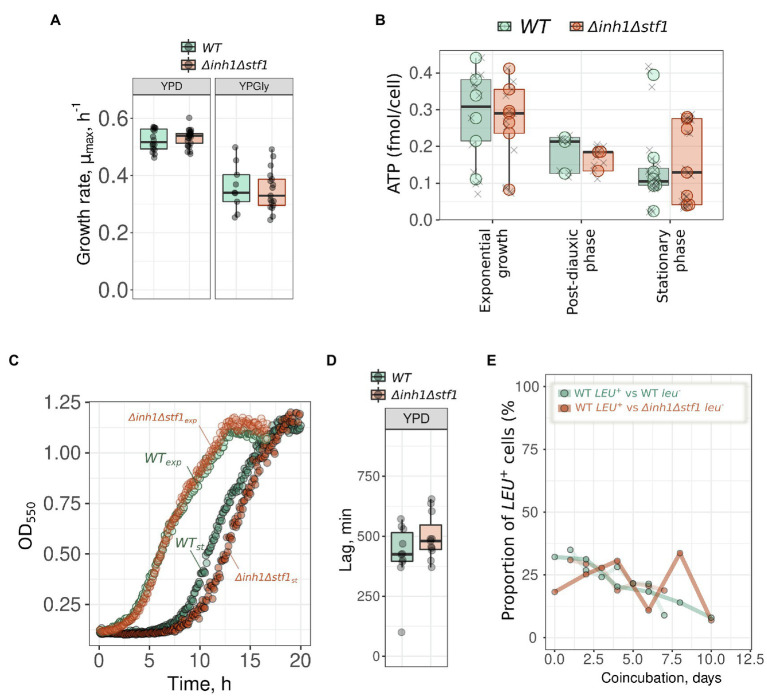
Double deletion of *INH1* and *STF1* has no effect on yeast cells fitness. **(A)** Growth rates (μ_max_) of the control *HIS ^+^ TRP^+^* (*WT*) and *Δinh1Δstf1* strains in rich medium supplemented with glucose (YPD) or glycerol (YPGly); **(B)** ATP concentration in the control *HIS ^+^ TRP ^+^ * (WT) and *Δinh1Δstf1* exponential, post-diauxic and stationary phases. Each circle data point represents an average of three ATP level measurements (individual values marked by grey crosses) in a separate day experiment. **(C)** Representative growth curves of yeast cells inoculated from the exponential (WT_exp_ and *Δinh1Δstf1_exp_*) or 10-days stationary phase (WT_st_ and *Δinh1Δstf1_st_*) into fresh YPD medium and **(D)** Quantification of the lag periods (in minutes) measured as time from the inoculation to the moment when yeast culture reached maximum growth rate. **(E)** Relative fitness of *Δinh1Δstf1* yeast cells in a competitive assay experiment. The proportion of *LEU*^+^ cells in the suspension was calculated from CFU numbers after plating suspensions on selective mediums (see the section Materials and Methods, *n* = 2).

Next, we compared the growth characteristics of *Δinh1Δstf1* (*rho^+^*) and control *HIS ^+^ TRP^+^* (*rho^+^*) strains. No differences in maximal growth rate μ_max_ were observed under conditions favoring glycolysis (YPD) or OxPhos (YPGly, Figure 4A). Then, we measured ATP levels in yeast cells that were collected from exponential, post-diauxic, and stationary growth phases. We calculated the ATP amount per cell using flow cytometry to assess the cell number in the samples. Although ATP levels decreased in post-diauxic and stationary phases, we did not detect significant differences between control and mutant strains (Figure 4B).

### *INH1* and *STF1* Double Deletion Shows No Pronounced Phenotype at the Level of Cell Suspension

The experimental results in Figure 2B indicated an earlier start of proliferation in the wild type strain compared to the *Δinh1Δstf1* mutant. We measured the time it took for *Δinh1Δstf1* and control yeast cells to reach their maximum growth rate (lag period) after inoculation into a fresh medium from the starved stationary phase culture. However, the difference in the lag period values between WT and *Δinh1Δstf1* was insignificant: *p* value according to Mann–Whitney U test was equal to 0.2 (Figures 4C,D). We also did not detect a difference in the number of colony-forming units in *Δinh1Δstf1* and wild type yeast suspensions during starvation in the stationary phase (Supplementary Figure S7).

Next, we performed a competitive assay experiment in which *Δinh1Δstf1* and wild type strains were grown together in the same flask. Such experiments can detect minor beneficial effects that are indistinguishable by growth rate measurements. Suspension of wild type *leu^−^* and *Δinh1Δstf1 LEU^+^* cells was passed through daily cycles of growth up to the post-diauxic phase, and then we transferred small aliquots of the suspension mix into the fresh medium. As a control, we compared changes in the proportion of *LEU*^+^ cells during co-cultivation of wild type *leu^−^* and wild type *LEU^+^* cells. In two experiments lasting at least 6 days (six cycles), we detected a decreasing trend of *LEU*^+^ cells proportion but no effect of *INH1* and *STF1* double deletion was found (Figure 4E).

We also compared yeast growth rates in the presence of protonophores. We expected that protonophores would induce mitochondrial depolarization, make F_O_F_1_ switch from ATP synthesis to hydrolysis and therefore suppress the growth of *Δinh1Δstf1* mutant but not of the wild type cells. We tested three anionic protonophores, FCCP, niclosamide (NCA), and pentachlorophenol (PCP). These compounds inhibited growth in the glycerol-based medium and increased the rate of oxygen consumption in yeast cells (Galkina et al., 2020a). It should be noted that the concentration of protonophores required for stimulation of respiration in intact cells is about an order of magnitude higher (Galkina et al., 2020a) than the concentration stimulating respiration of isolated mitochondria (Galkina et al., 2020b). However, in line with our results with starved yeast cells, we did not detect any difference in the maximal growth rate μ_max_ between control and *Δinh1Δstf1* strains (Supplementary Figure S8).

We also assessed the survival rate of *Δinh1Δstf1* and control strains under various stressful conditions: elevated temperature, high osmolarity, oxidative stress, and high concentrations of ethanol, but in all these experiments we did not detect a decrease in survival of *Δinh1Δstf1* strain (Supplementary Figure S9). Finally, we did not detect a decrease in *Δinh1Δstf1* resistance to acetic acid (Supplementary Figure S10), which is known to acidify mitochondrial matrix and inhibit the respiratory chain (Chaves et al., 2021).

## Discussion

Inhibition of F_O_F_1_ ATP-synthase by IF_1_ was characterized in detail on the biochemical level a long time ago (see reviews Schwerzmann and Pedersen, 1986; Rouslin, 1987; Hashimoto et al., 1990). It was established that the binding stoichiometry of IF_1_ and F_O_F_1_ is 1:1, that the binding occurs under conditions when the enzyme hydrolyzes ATP, and that upon membrane energization IF_1_ is released from the enzyme and ATP synthesis restarts. On a cellular level, IF_1_ protects cultivated animal cells from certain kinds of stress. It was found that lowering the amount of IF_1_ resulted in increased cell death in glucose-free medium during anoxia (Campanella et al., 2008). Similar effect was observed when cells were exposed to 2-deoxyglucose and cyanide so that both glycolysis and OxPhos were inhibited (Fujikawa et al., 2012). These results were explained by faster cellular ATP depletion occurring when IF_1_ was not inhibiting the ATPase activity of F_O_F_1_.

However, on the level of whole organisms, the role of IF_1_ is still unclear. No phenotype was found in mice with IF_1_ knockout (Nakamura et al., 2013). In nematode *Caenorhabditis elegans*, IF_1_ homolog was non-essential under normal physiological conditions but was found to increase survival under stresses induced by paraquat, cyanide, protonophore FCCP, and heat shock (Fernández-Cárdenas et al., 2017). In unicellular organisms, phenotypes of IF_1_ depletion are characterized at the level of isolated mitochondria or suggest the detrimental function of IF_1_.

Free-living microorganisms spend most of their time under the conditions of energy limitation (Lever et al., 2015). When the substrate becomes available, the rapid transition from growth arrest to proliferation becomes a crucial advantage in the competition for substrates. Therefore, microbial cells should maintain functional energy conversion machinery and be ready to start producing ATP for anabolic processes without delay. In batch cultures, when the glucose present in the medium is exhausted, yeast cells enter the post-diauxic phase. In this phase, yeast cells use non-fermentable carbon sources accumulated during exponential growth, such as ethanol. In several days, after ethanol is depleted, yeast cells enter the stationary phase (Herman, 2002).

It should be noted that glycolysis intermediate fructose-1,6-bisphosphate inhibits yeast respiration (Díaz-Ruiz et al., 2008). Therefore, post-diauxic and stationary phase cells inoculated into glucose-containing medium are expected to harbor inhibited respiration but active F_O_F_1_. Here, we suggested that ATPase inhibitory protein Inh1p in starved yeast cells could help them maintain a high level of functional F_O_F_1_ ATP-synthase while not wasting ATP due to its ATPase activity and enabling rapid growth recovery after starvation. In line with our suggestion, we found that post-diauxic and stationary-phase yeast cells accumulate high levels of Inh1p and Stf1p (Figure 1, Supplementary Figures S3, S4, S5).

Starving yeast cells differentiate into quiescent and non-quiescent cells. Quiescent (Q-) cells do not proliferate and are stress-resistant, whereas non-quiescent (NQ-) cells continue to proliferate but are susceptible to stresses (Aragon et al., 2008). Systematic analysis of yeast protein GFP fusions revealed that Q-cells usually have higher concentrations of mitochondrial proteins, including Inh1-GFP, if compared to NQ-cells (Davidson et al., 2011). Therefore, it is likely that in our experiments, Inh1p-positive cells represent the Q-cell subpopulation. Indeed, Figure 2B shows that Inh1p-positive cells at the beginning of the experiment have a low budding index. Moreover, we showed that high concentration of Inh1p-GFP in yeast cells correlates with their chances of budding shortly after the glucose was added to the medium (Figure 2).

At the same time, the effect of double deletion of *INH1* and *STF1* on yeast recovery from the stationary phase was marginal (Figures 4C–E). This seeming contradiction with the effect on budding can be explained in several ways. First, *INH1* expression can be correlated with an early exit from the stationary phase but not directly (causatively) contribute to the growth restart. Indeed, some other proteins accumulated in Inh1p-positive cells may limit the stationary phase exit. Second, given that yeast cells in dense cultures can communicate with each other (Hlavácek et al., 2009), the effect of ATPase protein inhibitors on growth recovery might be pronounced only in heterogeneous populations. We also cannot exclude the possibility that the effects of *INH1* on the recovery from starvation to growth are specific to the genetic background and genetic markers.

It should be noted that the strain with double deletion of *INH1* and *STF1* can rely on other mechanisms preventing ATP depletion. While IF_1_ inhibits ATPase activity of F_O_F_1_ ATPase upon mitochondrial matrix acidification, ADP can also lock the enzyme in the inactive state under de-energized conditions (Galkin and Vinogradov, 1999, see also Lapashina and Feniouk, 2018 for a recent review). This mechanism is conserved in eukaryotic mitochondria, chloroplasts and bacteria although the strength of the effect may vary between species (Lapashina and Feniouk, 2019; Lapashina et al., 2019). Therefore, ADP-inhibition of mitochondrial ATPase activity appears to be an inherent mechanism preventing excessive ATP hydrolysis under the conditions of mitochondrial depolarization. The redundancy of ADP-inhibition and IF_1_-mediated inhibition of ATP hydrolysis can mask the manifestations of IF_1_ deletion on the level of whole organisms including yeast. Therefore, we speculate that IF_1_ might appear to be crucial under the conditions when ADP-inhibition is ineffective, e.g., upon the depletion of adenine nucleotide pool in the cells.

To summarize, here we have shown that ATPase inhibitor proteins accumulate in starved yeast cells. Moreover, clonal suspensions of *BY4741* yeast strains show natural heterogeneity in IF_1_ level. Upon the addition of the fermentable carbon source, yeast cells with high mitochondrial concentration of IF_1_ were able to start budding more rapidly than cells lacking IF_1_. These observations suggest that IF_1_ plays an important role during starvation. However, given that the double deletion of *INH1* and *STF1* genes did not produce a pronounced effect, we suggest that the function of IF_1_ is partially redundant.

## Data Availability Statement

The raw data supporting the conclusions of this article will be made available by the authors, without undue reservation.

## Author Contributions

BF and DK designed the research and supervised the project. BF obtained the funding. KG, VZ, and NK generated and verified mutant strains. KG performed flow-cytometry and microscopy experiments and performed competition assay experiments. KG, OM, and VZ isolated mitochondria. AL and VZ measured ATP levels in yeast cells and ATP hydrolysis rates. KG and VZ measured yeast growth and survival. KG, AL, and DK prepared the illustrations. DK drafted the text. KG, NK, AL, and BF substantially edited the text. KG, VZ, NK, AL, OM, BF, and DK contributed to the conceptualization and text editing. All authors contributed to the article and approved the submitted version.

## Funding

This work was supported by the Russian Science Foundation (project 20-14-00268).

## Conflict of Interest

The authors declare that the research was conducted in the absence of any commercial or financial relationships that could be construed as a potential conflict of interest.

## Publisher’s Note

All claims expressed in this article are solely those of the authors and do not necessarily represent those of their affiliated organizations, or those of the publisher, the editors and the reviewers. Any product that may be evaluated in this article, or claim that may be made by its manufacturer, is not guaranteed or endorsed by the publisher.
