# Supplementary material for: Heterogeneity of Starved Yeast Cells in IF1 Levels Suggests the Role of This Protein in vivo
Source: Front Microbiol. 2022 Mar 23;13:816622. doi: 10.3389/fmicb.2022.816622 (PMC8984185; doi:10.3389/fmicb.2022.816622)
Supplement: Supplementary file 1 [file Data_Sheet_1.pdf]

# Supplementary materials:

# Heterogeneity of starved yeast cells in $IF_1$ levels suggests the role of this protein *in vivo*

Galkina K.V.[1,2], Zubareva V.M.[2], Kashko N.D.[2], Lapashina A.S.[1,2,3], Markova O.V.[1], Feniouk B.A.[1,2], Knorre D.A.[1,2]

[1]*A.N. Belozersky Institute of Physico-Chemical Biology, Lomonosov Moscow State University, Moscow, Russia*
[2]*Faculty of Bioengineering and Bioinformatics, Lomonosov Moscow State University, Moscow, Russia*
[3]*Department of Biological Chemistry, Sechenov First Moscow State Medical University, Moscow, Russia*

**Table S1. Strains used in this study**

| **Name** | **Genotype** | **Reference/ Way of making** | **Parental strain** |
|---|---|---|---|
| *W303-1A* | *MATa ade2-101 his3-11 trp1-1 ura3-52 can1-100 leu2-3 rho$^+$* | *Laboratory of A. Hyman* | |
| *HIS+TRP+* | *MATa ade2-101 his3-11 trp1-1 ura3-52 can1-100 leu2-3 HIS3 TRP1 rho$^+$* | Strain produced by the transformation of the PCR cassette | *W303-1A* |
| *HIS+TRP+ [rho$^0$]* | *MATa ade2-101 his3-11 trp1-1 ura3-52 can1-100 leu2-3 HIS3 TRP1 [rho$^0$]* | Strain produced by incubation with ethidium bromide | *HIS+TRP+* |
| *Δstf1 TRP+* | *MATa ade2-101 his3-11 trp1-1 ura3-52 can1-100 leu2-3 stf1Δ::TRP1* | Strain produced by the transformation of the PCR cassette | *W303-1A* |

| | | | |
|---|---|---|---|
| | *$rho^+$* | | |
| *Δinh1 TRP+* | *MATa ade2-101 his3-11 trp1-1 ura3-52 can1-100 leu2-3 inh1Δ::TRP1 $rho^+$* | Strain produced by the transformation of the PCR cassette | *W303-1A* |
| *Δstf1 HIS+* | *MATa ade2-101 his3-11 trp1-1 ura3-52 can1-100 leu2-3 stf1Δ::HIS3 $rho^+$* | Strain produced by the transformation of the PCR cassette | *W303-1A* |
| *Δinh1 HIS+* | *MATa ade2-101 his3-11 trp1-1 ura3-52 can1-100 leu2-3 inh1Δ::HIS3 $rho^+$* | Strain produced by the transformation of the PCR cassette | *W303-1A* |
| *Δinh1Δstf1* | *MATa ade2-101 his3-11 trp1-1 ura3-52 can1-100 leu2-3 inh1Δ::HIS3 stf1Δ::TRP1 $rho^+$* | Strain produced by the transformation of the PCR cassette | *Δstf1 TRP+* |
| *Δinh1Δstf1 $rho^0$* | *MATa ade2-101 his3-11 trp1-1 ura3-52 can1-100 leu2-3 inh1Δ::HIS3 stf1Δ::TRP1 [$rho^0$]* | Strain produced by incubation with ethidium bromide | *Δinh1Δstf1* |
| *BY4741* | *MATa his3Δ1 leu2Δ0 met15Δ0 ura3Δ0 $rho^+$* | (Huh et al. 2003) | |
| *Inh1-GFP* | *MATa his3Δ1 leu2Δ0 met15Δ0 ura3Δ0 INH1-GFP::HIS3 $rho^+$* | (Huh et al. 2003) | |
| *Stf1-GFP* | *MATa his3Δ1 leu2Δ0 met15Δ0 ura3Δ0 STF1-GFP::HIS3 $rho^+$* | *(Huh et al. 2003)* | |
| *Inh1-GFP W303* | *MATa ade2-101 his3-11 trp1-1 ura3-52 can1-100 leu2-3 INH1-GFP::HIS3 $rho^+$* | Strain produced by the transformation of the PCR cassette | *W303-1A* |

| | | | |
|---|---|---|---|
| *Stf1-GFP W303* | *MATa ade2-101 his3-11 trp1-1 ura3-52 can1-100 leu2-3 STF1-GFP::HIS3 rho$^+$* | Strain produced by the transformation of the PCR cassette | *W303-1A* |
| *LEU+* | *MATa ade2-101 his3-11 trp1-1 ura3-52 can1-100 leu2-3Δ::LEU2 rho$^+$* | (Karavaeva et al. 2017) | |

**Table S2. Primers**

| Primer | Sequence |
| --- | --- |
| HIS3-F | 5’-TCCTTTCCCGCAATTTTCTT |
| HIS3-R | 5’-GACACGTATAGAATGATGCA |
| HIS3-test | 5’-TGCATCATTCTATACGTGTC |
| TRP1-F | 5’- ATGTCTGTTATTAATTTCAC |
| TRP1-R | 5’- CCTATTTCTTAGCATTTTTG |
| TRP1-test | 5’- CACGCCAACCAAGTATTTCG |
| INH1-F | 5’-ATTTTACACGCATTACTACAGCACACTTTTATACAGTTCCACAATA GAATCGGATCCCCGGGTTAATTAA |
| INH1-R | 5’-GTAAGAATATTCTAAAAAAAAAAAAAAAAAAAGCTTCTGCGGAAAC GCATGGAATTCGAGCTCGTTTAAAC |
| INH1-F-test | 5’ - GCCAATCAACTGTCTAGTTG |
| INH1-R-test | 5’ - CTGTTCCTATCTTTCTATGC |
| STF1-F | 5’-ACAAGAAAAAAATTAGAGAATTTAGAAAACAAAATTAATAACCTTTCA AAGCGGATCCCCGGGTTAATTAA |
| STF1-R | 5’-TTCTACGTACAAAACGTGTCTGCAGAACCTTTAGCCTTAACAATG ACCAAGAATTCGAGCTCGTTTAAAC |
| STF1-F-test | 5’ - GCTATTATACTCTCACGGAG |
| STF1-R-test | 5’ - TGATGTACGGATGTTTTGTG |
| INH1-GFP-F | 5’ - TAAAATTGACTCGATGACCA |
| INH1-GFP-R | 5’ - GACTATTGACGTTATTTGGA |
| STF1-GFP-F | 5’-ACAAGAAAAAAATTAGAGAATTTAGAAAACAAAATTAATAACCTTTCA AAGCGGATCCCCGGGTTAATTAA |
| STF1-GFP-R | 5’-TTCTACGTACAAAACGTGTCTGCAGAACCTTTAGCCTTAACAATG ACCAAGAATTCGAGCTCGTTTAAAC |
| RT-qPCR-INH1-F | 5’ - AGGTTCTACTCTGAAGGTT |
| RT-qPCR-INH1-R | 5’ - CTGCCTAACGAAGAAGTC |
| RT-qPCR-STF1-F | 5’ - ATGGTCCTCTCGGTGGTG |
| RT-qPCR-STF1-R | 5’ - TTGTTGTCTGGCATAATAATCCTCTT |

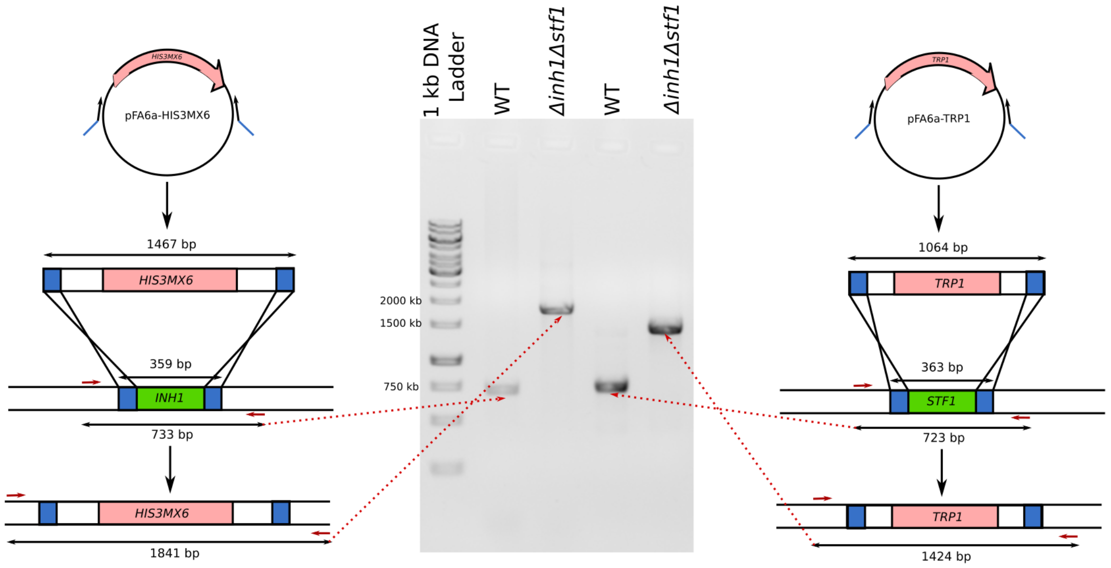


**Figure S1. Cloning and verification scheme of *INH1* and *STF1* deletion.** Clonings were conducted using disruption cassettes produced with PCR using pFA6a-HIS3MX6 or pFA6-TRP1 plasmids as matrices (Longtine et al. 1998). Primers are indicated in Table S2.

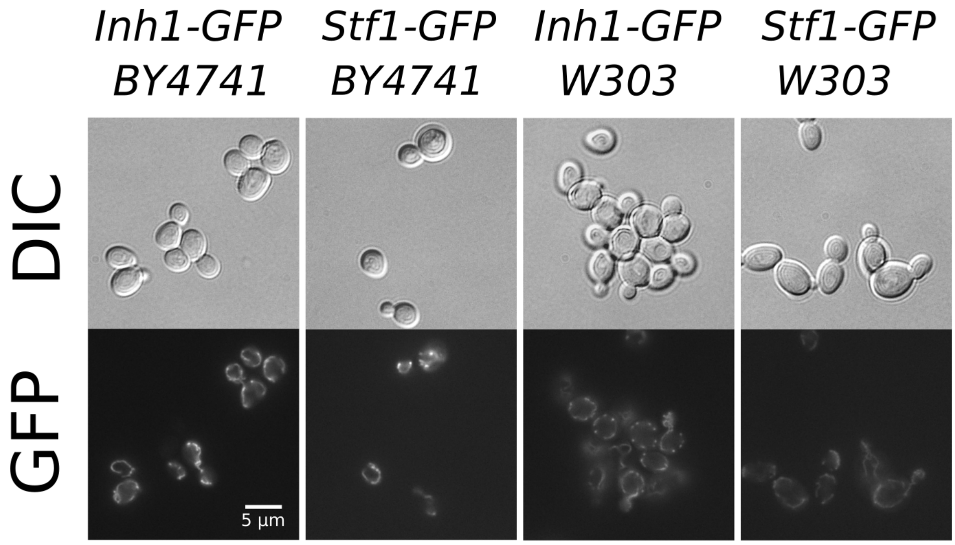


**Figure S2. Localization of GFP signal in Inh1-GFP and Stf1-GFP expressing cells. To make these photographs we took** yeast cells that were grown in YP raffinose up to exponential growth phase. DIC — differential interference contrast.

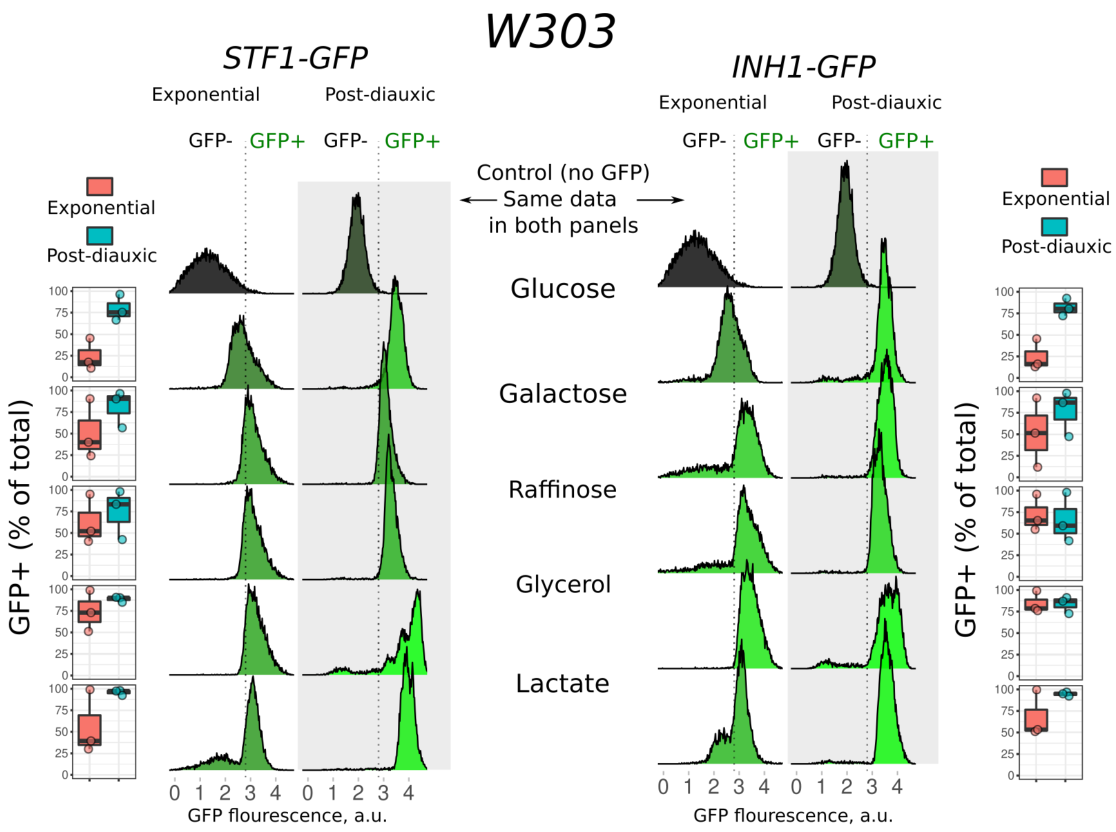


**Figure S3. Accumulation of Stf1-GFP (left panel) and Inh1-GFP (right panel) in exponentially growing and post-diauxic *W303-1A* yeast cells.** Boxplots show the proportion of the cells with GFP levels above the maximum value of autofluorescence. Histograms show the results of representative experiments; the upper panel shows the distribution of yeast cells autofluorescence in the parental control strain without GFP constructs.

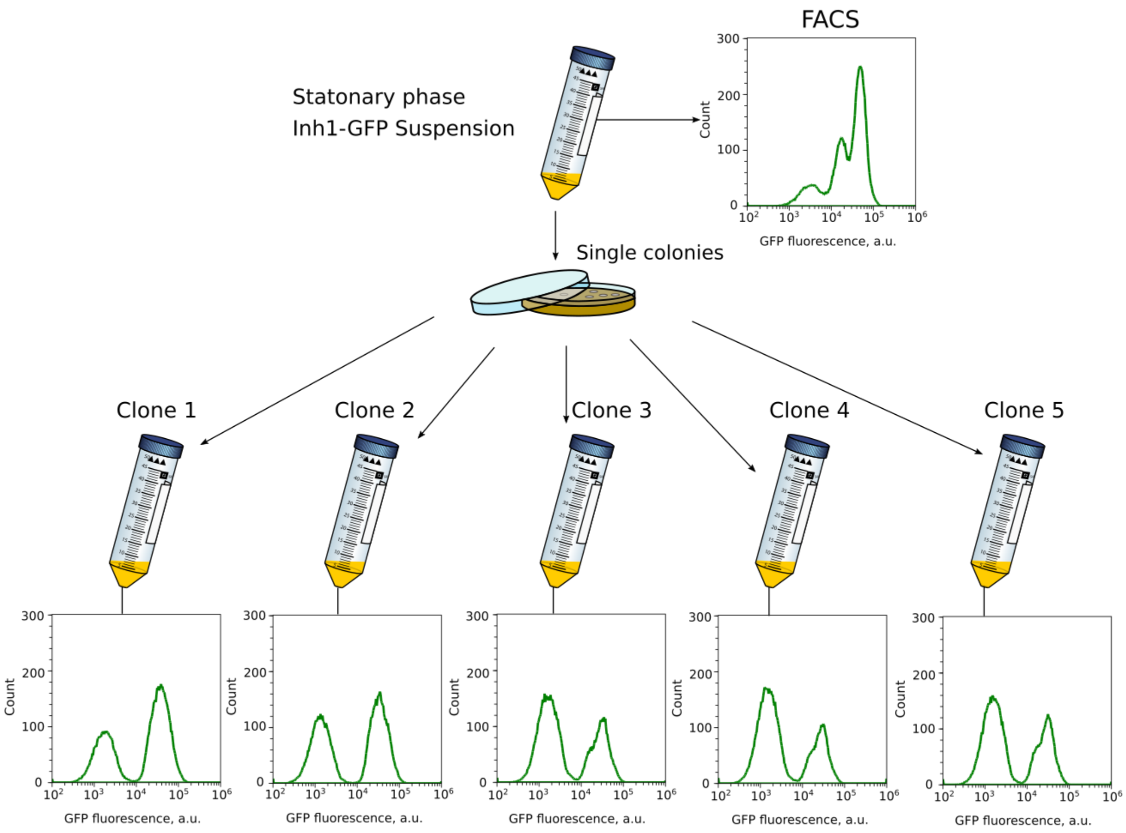


**Figure S4. Heterogeneity in the content of Inh1-GFP in stationary phase *BY4741* yeast cells.** Yeast strain *Inh1-GFP* was grown in YPD up to the post-diauxic phase and was dispersed to individual colonies on solid YPD media. Then we selected five random clones and incubated them in liquid YPD up to the stationary phase. We assessed Inh1-GFP heterogeneity in the initial suspension and in 5 monoclonal yeast cultures using flow cytometry.

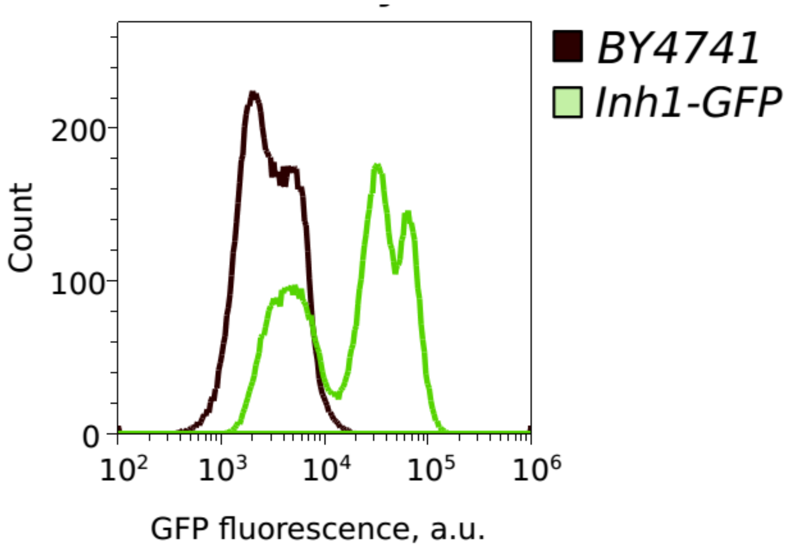


**Figure S5. Heterogeneity in the content of Inh1-GFP in yeast cells after 10 days incubation (stationary phase).**

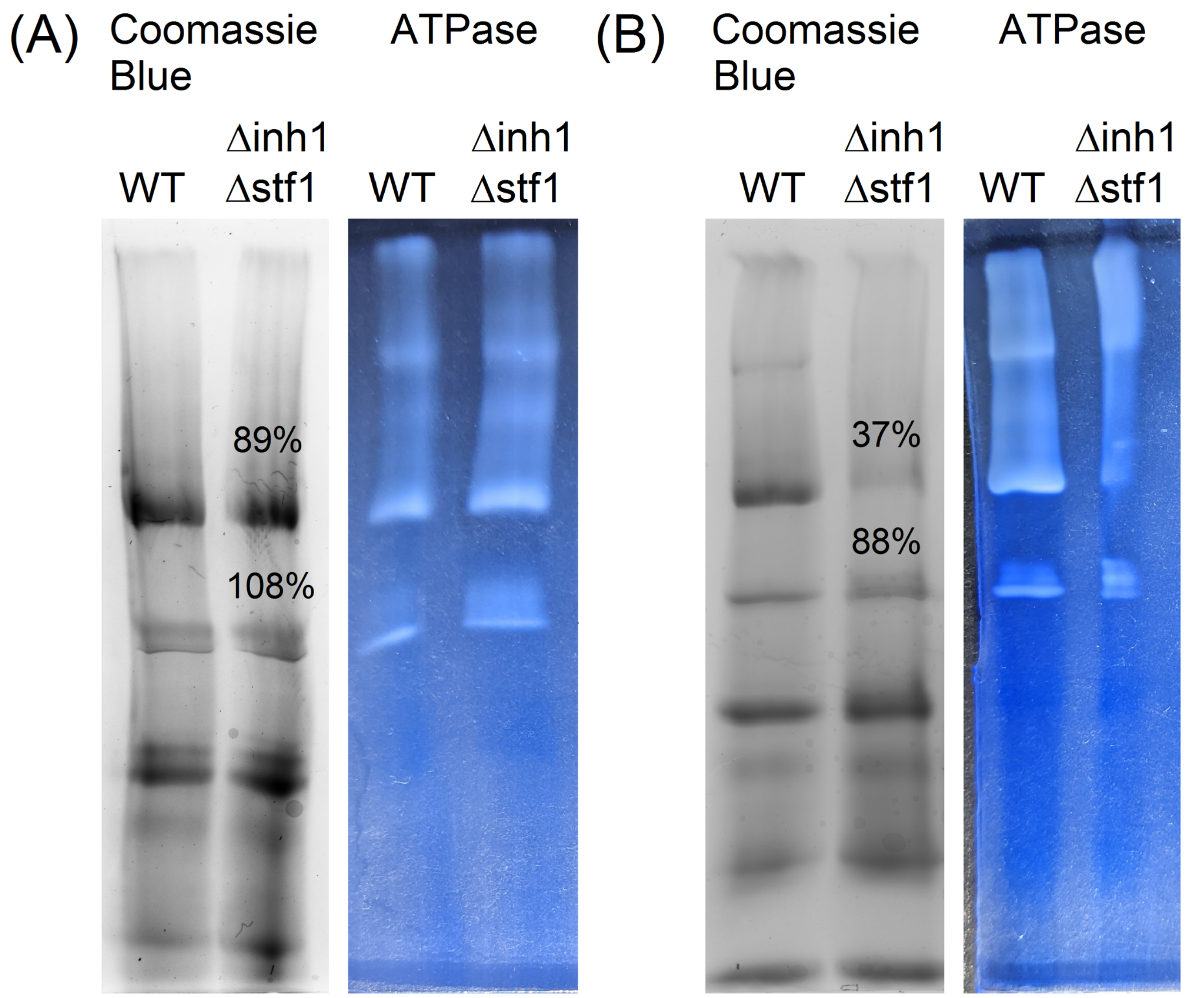


**Figure S6. BN-PAGE of solubilized mitochondrial complexes of control WT (*HIS*$^+$*TRP*$^+$) and mutant *Δinh1Δstf1* yeast strains.** Mitochondria were isolated as described in the Material and Methods section, two independent mitochondrial preparations of each strain (A and B) were analyzed. We solubilized mitochondria using dodecylmaltoside (detergent/protein ratio 0.5 g/g) according to (Wittig, Braun, and Schägger 2006). The wells contained either ~100 µg of protein/well (equalized for both preparations) for semi-quantitative analysis of protein-complexes concentration (Coomassie blue staining), or ~10 µg protein for in-gel ATPase activity detection. Electrophoresis was performed in 4-10% (A) or 4-12% (B) gradient acrylamide gels as described in (Wittig, Braun, and Schägger 2006). After the run, the gel was either stained with Coomassie Blue G-250 dye or assayed for ATPase activity according to (Timón-Gómez et al. 2020). In Coomassie-stained gels, bands corresponding to those demonstrating ATPase activity were assumed to be either full $F_OF_1$ ATP synthase complex or its $F_1$ portion. These bands were compared between *Δinh1Δstf1* and WT strains by densitometry. Percentages in the figure indicate the *Δinh1Δstf1* band size normalized by the corresponding value (100%) of the control (WT) sample.

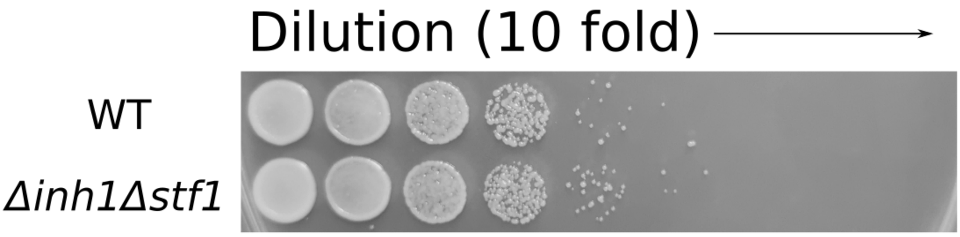


**Figure S7. Double deletion of *INH1* and *STF1* does not decrease the survival of yeast cells in the stationary phase.** Colonies formed by yeast wild type and *Δinh1Δstf1* suspensions incubated for eight days in YPD.

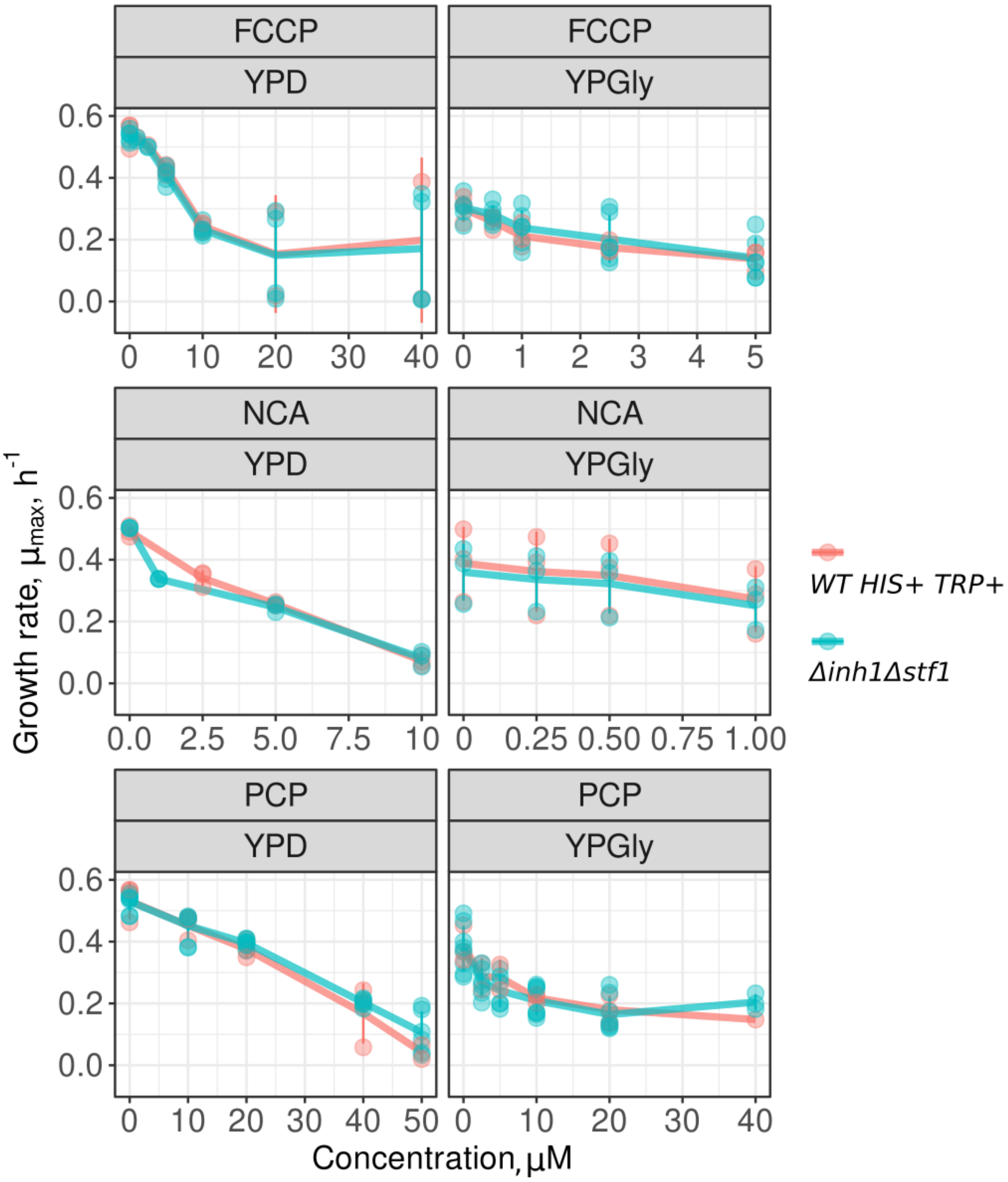


**Figure S8. Maximal growth rates of the *Δinh1Δstf1* and control strains (*WT TRP+ HIS+*) in the presence of three tested protonophores, carbonyl cyanide-p-trifluoromethoxy phenylhydrazone (FCCP), niclosamide (NCA) or pentachlorophenol (PCP).**

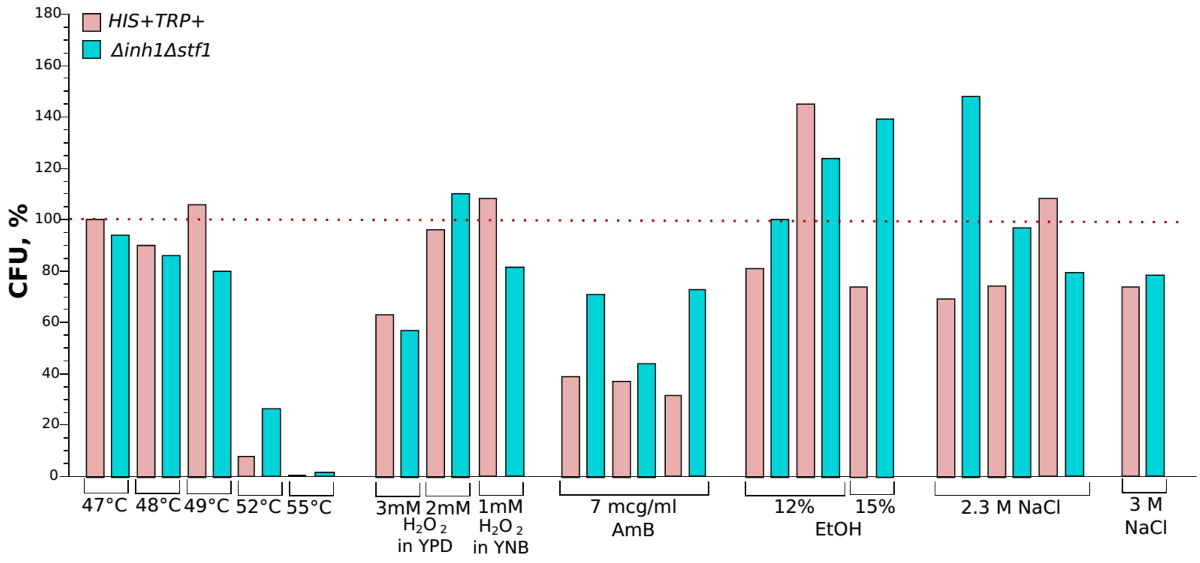


**Figure S9. Survival of WT (*HIS*⁺*TRP*⁺) and mutant *Δinh1Δstf1* strains under various stressful conditions**. Each bar represents the result of a single experiment. Yeast cells were grown in batch cultures up to the post-diauxic phase and then were incubated for 3 hours under indicated stress conditions. In Y-axis, 100% colony forming units (CFU) represents the number of colonies formed by the same yeast suspension spotted on agar before the stress. Values above 100% indicate that under selected stress conditions CFU increased.

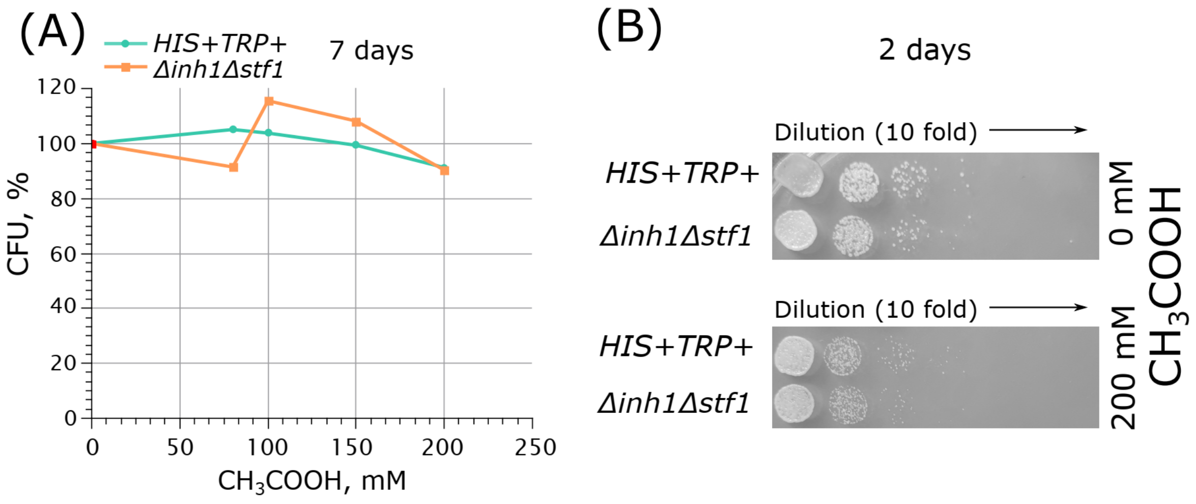


**Figure S10. Control (*HIS*⁺*TRP*⁺) and mutant *Δinh1Δstf1* strains show no difference in resistance to acidification of cytosol induced by acetic acid.** Survival of (A) stationary phase and (B) post-diauxic phase yeast cells.

### References for Supplementary Materials


Huh, Won-Ki, James V. Falvo, Luke C. Gerke, Adam S. Carroll, Russell W. Howson, Jonathan S. Weissman, and Erin K. O'Shea. 2003. "Global Analysis of Protein Localization in Budding Yeast." *Nature* 425 (6959): 686–91.

Karavaeva, Iuliia E., Sergey A. Golyshev, Ekaterina A. Smirnova, Svyatoslav S. Sokolov, Fedor F. Severin, and Dmitry A. Knorre. 2017. "Mitochondrial Depolarization in Yeast

Zygotes Inhibits Clonal Expansion of Selfish mtDNA.” *Journal of Cell Science* 130 (7): 1274–84.
Longtine, M. S., A. McKenzie 3rd, D. J. Demarini, N. G. Shah, A. Wach, A. Brachat, P. Philippsen, and J. R. Pringle. 1998. “Additional Modules for Versatile and Economical PCR-Based Gene Deletion and Modification in Saccharomyces Cerevisiae.” *Yeast* 14 (10): 953–61.
Timón-Gómez, Alba, Rafael Pérez-Pérez, Eva Nyvltova, Cristina Ugalde, Flavia Fontanesi, and Antoni Barrientos. 2020. “Protocol for the Analysis of Yeast and Human Mitochondrial Respiratory Chain Complexes and Supercomplexes by Blue Native Electrophoresis.” *STAR Protocols* 1 (2). https://doi.org/10.1016/j.xpro.2020.100089.
Wittig, Ilka, Hans-Peter Braun, and Hermann Schägger. 2006. “Blue Native PAGE.” *Nature Protocols* 1 (1): 418–28.
